# Influence of gut microbiota on mucosal IgA antibody response to the polio vaccine

**DOI:** 10.1038/s41541-020-0194-5

**Published:** 2020-06-09

**Authors:** Ting Zhao, Jing Li, Yuting Fu, Hui Ye, Xiaochang Liu, Guoliang Li, Xiaolei Yang, Jingsi Yang

**Affiliations:** Yunnan Key Laboratory of Vaccine Research and Development on Severe Infectious Disease, Institute of Medical Biology, Chinese Academy of Medical Sciences and Peking Union Medical College, Kunming, Yunnan China

**Keywords:** Immunology, Microbiology, Health care

## Abstract

The impact of intestinal microbiota on mucosal antibody response to the polio vaccine is poorly understood. We examined changes in vaccine-induced intestinal mucosal immunity to poliovirus by measuring the immunoglobulin A (IgA) antibody levels in stool samples collected from 107 infants in China, and the samples were collected 14 days after different sequential vaccinations combining inactivated polio vaccine (IPV) with oral poliovirus vaccine (OPV). Gut microbiota were identified using 16S ribosomal RNA sequencing 28 days before, 14 days before, and at the last dose of OPV. Vaccine-induced type 2-specific mucosal IgA showed a decrease after switching from trivalent to bivalent OPV (bOPV) (positive rate of polio type 2-specific mucosal IgA, 16.7%, 11.8%, and 45.9% for IPV + 2bOPV, 2IPV + bOPV, and 2IPV + trivalent OPV groups, respectively). The composition of the gut microbiome was significantly different, a higher abundance of *Firmicutes* and a lower abundance of *Actinobacteria* were observed in IgA-negative infant (*n* = 66) compared with IgA-positive infants (*n* = 39), and the gut microbiota were more diverse in IgA-negative infants on the day of OPV inoculation. The abundance of *Clostridia* was concomitant with a significantly lower conversion rate of mucosal IgA responses to the polio vaccine. The composition of the gut microbiome may affect the intestinal mucosal IgA response to the polio vaccine.

## Introduction

The Global Polio Eradication Initiative has reached a new stage. Wild poliovirus type 2 (PV2) was eliminated worldwide in 1999, and no cases of wild PV3 have been reported since the end of 2012. Although the number of cases caused by wild poliovirus has decreased dramatically, the Sabin-attenuated strains originating from the oral poliovirus vaccine (OPV) have the potential to regain their neurovirulence and transmission characteristics, leading to vaccine-associated paralytic poliomyelitis (VAPP) and circulating vaccine-derived poliovirus (cVDPV)^1^.

To overcome VAPP and cVDPV, after April 2016, Sabin 2 from the trivalent oral polio vaccine (tOPV) was removed, and at least one dose of inactivated polio vaccine (IPV) was introduced. Compared with IPV, OPV not only induces poliovirus-specific serum antibodies, but also induces a high level of intestinal immunity to prevent the fecal–oral spread of poliovirus, which is the primary transmission route. Poliovirus-specific immunoglobulin A (IgA) is considered a likely correlate of preventing excretion of poliovirus, and IPV was found not to induce secretion of IgA in the intestine^2^. Several studies indicated that after one-dose challenge with monovalent OPV type 2, only a few infants who had received three doses of tOPV showed viral shedding. By contrast, more than half of the infants who had received three doses of bivalent OPV (bOPV) or bOPV–IPV showed viral shedding. Mucosal type 2-specific antibodies can be induced in infants who receive OPV type 2, thereby influencing viral shedding^3^. As a strategy for eliminating VAPP and cVDPV, reduced use of live-attenuated poliovirus vaccines is necessary. However, the enhancement of vaccine mucosal immunity warrants further attention.

Recently, a few studies^4–11^ have shown that children in poorer regions with poor sanitation induce lower immune responses compared with children in developed regions. The OPV has not solved the issue of impaired vaccine immunogenicity in poor-sanitation regions^12,13^. Vaccine trials have reported that immune responses to OPV in developing countries are suboptimal, and in these regions, seroconversion rates are inferior to the near-complete seroconversion rates observed in high-income countries. Despite decades of research, we have failed to establish a clear understanding of the influence of poor sanitation on vaccine effectiveness^11,13^.

Although the mechanisms underlying this phenomenon remain unknown, some studies found a significant difference in the microbiota compositions between people living in developed and developing regions^14–16^. A hypothesis was therefore posed that populations in poor-sanitation regions are exposed to a larger variety of harmful microorganisms resulting in an altered intestinal immune system and dysbiotic microbiota. Oral vaccine antibody responses may fail to be triggered in children with intestinal microbial dysbiosis^17^.

Thus, researchers began to examine the relationship between the microbiome and vaccine effectiveness. A study in Bangladesh showed that the phylum *Actinobacteria* is positively correlated with the OPV-induced IgG response and the CD4^+^ T cell response^18^. Another study in South India did not detect a significant difference in bacterial taxa between OPV responders and OPV nonresponders, but the diversity index of the microbiota for nonresponders was higher^19^. Moreover, one study conducted on Indian infants revealed that azithromycin did not improve the immunogenicity of the OPV despite reducing the biomarkers of environmental enteropathy^20^.

Following the above studies, which examined whether neutralizing antibody responses activated by the poliovirus vaccine were associated with specific bacterial microbiota^18–20^, our study investigated gut mucosal IgA response to the polio vaccine in infants with different sequential immunization programs. We further analyzed the relationship between the composition of intestinal microbiota and gut mucosal response to the polio vaccine to better understand the factors that affect the intestinal mucosal immune response.

## Results

### Vaccine-induced mucosal immunity to poliovirus

**Fig. 1:**
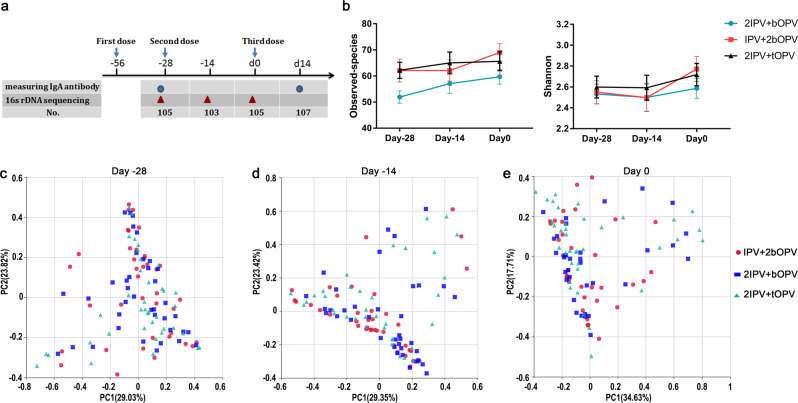
α- and β-Diversity of gut microbiota in different immunization schedules. **a** Study design. The blue circle represents the measurement of IgA antibody levels from stool samples; red triangles represent the assessment of bacterial microbiota composition by sequencing the 16S rRNA gene V4 region; and no. in the column lists represents the number of detections. The first, second, and third doses of vaccine were inoculated at the age of 2 (day −56), 3 (day −28), and 4 (day 0) months, respectively. **b** Observed species and Shannon index (presented as the means with SEM) from the three immunization schedules groups at days −28 (*n* = 105), −14 (*n* = 103), and 0 (*n* = 105). **c** Weighted UniFrac principal coordinate analysis of the bacterial microbiota composition from the three immunization schedules groups on day −28 (*n* = 105), day −14 (*n* = 103), and day 0 (*n* = 105).

We performed a random, double-blind trial in GuangXi, China. In our study, 1200 healthy 2-month-old infants were recruited and randomly assigned to three different sequential immunization schedules combining IPV and OPV: IPV + 2bOPV, 2IPV + bOPV, and 2IPV + tOPV, receiving vaccines at ages 2, 3, and 4 months (corresponding to day −56, −28, and 0, respectively, in Fig. 1a). At 2 weeks after the third dose of poliovirus vaccine immunization, vaccine-induced mucosal immunity was analyzed by measuring IgA antibody levels of stool samples in 107 of 120 infants vaccinated with three different sequential immunizations: IPV + 2bOPV, 2IPV + bOPV, or 2IPV + tOPV, which represented ~10% of the infants in the above trial, 13 infants were lost. The demographic characteristics (sex, race, and feeding status) in three different sequential immunizations are shown in Supplementary Table 1, and there was no significant difference among the three groups. Because IPV is the first dose of vaccine, and in general IPV does not generally induce poliovirus shedding and mucosal immunity, stool samples were not collected before or after the first dose of inoculation (day −56) according to the clinical protocol. We started collecting stool samples before the second dose (day −28), and none of the participants had been vaccinated with OPV before the second dose. Therefore, stool samples before the second dose (day −28) can be regarded as a pre-immunization control, and the number of infants that were IgA positive at baseline is shown in the Supplementary Table 1. No significant difference was detected between the three groups at baseline. Few infants were positive of polio-specific IgA, and this may be due to maternal antibodies or exposure to the Sabin-attenuated strain. The different immunization schedules had no apparent differences in effect on vaccine-induced polio-specific mucosal IgA antibody immunity (Table 1). However, the conversion rate of type 2-specific IgA in stools was significantly higher in infants administered 2IPV + tOPV than in those receiving IPV + 2bOPV or 2IPV + bOPV. Furthermore, no significant differences were observed in the type 1-specific IgA and type 3-specific IgA conversion rates in each type of sequential immunization (Table 1).

**Table 1:** Polio-specific mucosal IgA measured in stool samples collected from infants 2 weeks after the last dose of vaccination.

	Total no	Proportion with detectable polio-specific IgA antibody							
		PV-IgA, no. (%)	*P* value, *χ*^2^ rest	PV1-IgA, no. (%)	*P* value, *χ*^2^ test	PV2-IgA, no. (%)	*P* value, *χ*^2^ test	PV3-IgA, no. (%)	*P* value, *χ*^2^ test
Schedule			0.175		0.646		0.001		0.592
IPV + 2bOPV	36	24 (66.7%)		14 (38.9%)		6 (16.7%)		12 (33.3%)	
2IPV + bOPV	34	17 (50%)		12 (35.3%)		4 (11.8%)		9 (26.5%)	
2IPV + tOPV	37	26 (70.3%)		17 (45.9%)		17 (45.9%)		14 (37.8%)	
Sex			0.902		0.451		0.864		0.883
Male	57	36 (63.2%)		21 (36.8%)		14 (24.6%)		19 (33.3%)	
Female	50	31 (62%)		22 (44%)		13 (26%)		16 (32%)	
Race			0.901		0.481		0.497		0.957
Han	34	21 (61.8%)		12 (35.3%)		10 (29.4%)		11 (32.4%)	
Minorities	73	46 (63%)		31 (42.5%)		17 (23.3%)		24 (32.9%)	
Feeding			<0.001		0.062		0.326		0.012
Breastfeeding	71	53 (74.6%)		33 (46.5%)		2 (28.2%)		29 (40.8%)	
Formula feeding	36	14 (38.9%)		10 (27.8%)		7 (19.4%)		6 (16.7%)	

*tIPV* trivalent inactivated polio vaccine, *bOPV* bivalent oral polio vaccine, *PV* poliovirus, *PV1* poliovirus type 1, *PV2* poliovirus type 2, *PV3* poliovirus type 3.

We tested whether polio-specific intestinal mucosal IgA response is affected by sex, race, and feeding formula. To test whether sex, race, and feeding formula correlated with IgA-associated differences, the conversion rates of polio-specific intestinal IgA were conducted in boys and girls, those of Han and other minority ethnicities, and breast-fed and formula-fed infants, respectively (Table 1). We observed that sex (conversion rates of polio-specific intestinal IgA: 63.2% [male] versus 62% [female], *p* = 0.902, *χ*^2^ test) and race (conversion rates of polio-specific intestinal IgA: 61.8% [Han] versus 63% [other minorities], *p* = 0.901, *χ*^2^ test) were not significantly correlated with polio-specific IgA secretion. Performance of polio-specific IgA secretion between breast-fed and formula-fed infants was significantly different (conversion rates of polio-specific intestinal IgA: 74.6% [breast-fed] versus 38.9% [formula-fed], *p* < 0.001, *χ*^2^ test), and breastfeeding seems more beneficial to the conversion of poliovirus-specific IgA than formula feeding (Table 1). However, when we analyzed the different serotype groups, this difference was statistically significant for polio type 3-specific intestinal IgA, but not for polio type 1- or 2-specific intestinal IgA (Table 1). We further compared the microbiota compositon with the feeding status to examine whether breastfeeding or formula feeding significantly affected the intestinal microbiome. After comparison, we found that the α-diversity (observed species and Shannon index) and the main composition of the bacterial microbiota did not differ significantly between breast-fed and formula-fed infants (Supplementary Tables 2 and 3). Therefore, the results indicated that feeding status did not significantly affect the intestinal microbiota composition. The existing literature reports that breastfeeding affects the establishment of the intestinal microbiota in early childhood and is associated with higher levels of *Bifidobacterium* species and the suppression of *Firmicutes* species in infants^21^. However, we failed to observe a significant difference in the microbiota composition between formula-fed and breast-fed infants. There were several factors might account for that we failed to observe such a difference. According to the inclusion/exclusion criteria of our clinical trial, caregivers were not prohibited from administering probiotics to their infants; in China, probiotics are commonly given to infants as a supplement, which provides a source of live bacteria that assists in the early establishment of the intestinal microbiota. Furthermore, environmental factors (e.g., geographical location, household pets, family members) may alter associations between infant feeding and the intestinal microbiota.

### Association between the composition of the bacterial microbiota and immunization schedules

In total, 313 samples from infants were collected 28 days before (day −28, *n* = 105), 14 days before (day −14, *n* = 103), and on the day of the last dose of OPV (day 0, *n* = 105) (Fig. 1a). We assessed the composition of the bacterial microbiota by sequencing the V4 hypervariable region of the 16S ribosomal RNA (rRNA) gene in stool samples. We obtained 44,361 ± 7433 (mean ± s.d. [standard deviation]) reads per sample, and 735 operational taxonomic units (OTUs) with 64 ± 22 (mean ± s.d.) OTUs per sample were delineated using 97% as a homology cut-off value (Supplementary Fig. S1).

We did not observe a significant difference in observed species, Shannon index, relative taxon abundances, or weighted UniFrac distance among different immunization schedules (rarefaction depth: 18,512) (Fig. 1b, Supplementary Table 4). Principal coordinate analysis (PCoA) extracts major elements and structures that reflect the differences between samples to the greatest extent. Samples with high similarity in community structure tend to cluster together, while those with low similarity are located far apart^22^. PCoA showed that the structures of the gut microbiota in different immunization schedules were similar, and no significant immunization schedule-related shifts in the gut microbiota were observed in infants of the same age (Fig. 1c). These results suggest that the different sequential immunization schedule for the poliovirus vaccine had no significant effect on the clustering patterns of the gut microbiota.

### Association between intestinal mucosal IgA response to polio vaccine and the composition of the bacterial microbiota

To investigate the effect of bacterial microbiota characteristics on the OPV-induced IgA response, we collected data on the gut microbiota from fecal samples of infants before the last OPV inoculation (day −28, −14, and 0). The fecal samples were divided into two groups, according to whether the IgA response to any poliovirus serotype was positive or negative after OPV inoculation (day14): polio-specific IgA-positive (IgA.P) infants (day −28, *n* = 66; day −14, *n* = 65; day 0, *n* = 66) and polio-specific IgA-negative (IgA.N) infants (day −28, *n* = 39; day −14, *n* = 38; day 0, *n* = 39). All infants appeared healthy, and no fever, diarrhea, and other clinical symptoms were detected during sampling.

**Fig. 2:**
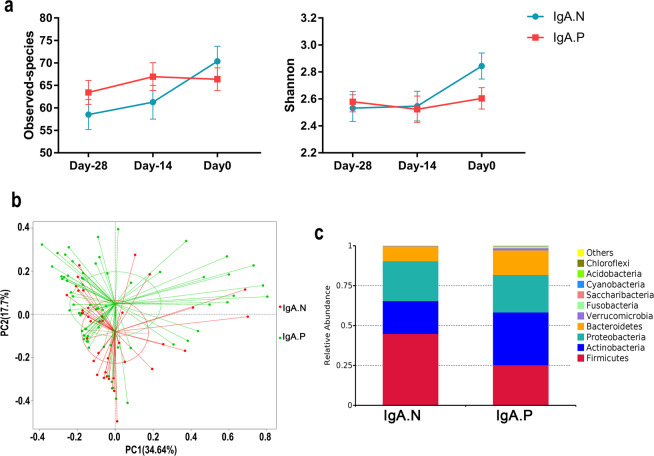
Association between intestinal mucosal IgA response to the polio vaccine and the composition of the bacterial microbiota. **a** Observed species count and Shannon index (presented as the means with SEM) from the IgA.N and IgA.P groups at day −28 (*n* = 66 for IgA.P, *n* = 39 for IgA.N), day −14 (*n* = 65 for IgA.P, *n* = 38 for IgA.N), and day 0 (*n* = 66 for IgA.P, *n* = 39 for IgA.N). **b** Weighted UniFrac principal coordinates analysis of the bacterial microbiota composition of IgA.N and IgA.P groups (*n* = 66 for IgA.P, *n* = 39 for IgA.N). **c** Phylum-level compositions of the bacterial microbiota from IgA.N or IgA.P groups at the time of the third dose vaccination (*n* = 66 for IgA.P, *n* = 39 for IgA.N).

We wished to determine whether gut microbiota diversity affected the induction of polio-specific IgA. The observed species and the Shannon index constitute the α-diversity used for analyzing community richness and diversity of the bacterial microbiota within a community^22^. At the time of receiving the OPV, a lower Shannon index was apparent in infants whose polio-specific IgA was positive after receiving the OPV (day 0, Fig. 2a, Table 2, Wilcoxon’s test, *p* = 0.0478). Similar trends were obtained when analyzing the observed species; however, this difference was not statistically significant (Fig. 2a, Table 2, Wilcoxon’s test, *p* = 0.3753).

**Table 2:** α- and β-Diversity of gut microbiota in IgA-positive and IgA-negative infants.

	Days before vaccination	IgA positive (*n* = 66)	IgA negative (*n* = 39)	*P* value	Test method
α-Diversity					
Observed species	Day −28	63.44 (58.11–68.77)	58.51 (51.72–65.3)	0.188	Wilcoxon’s test
	Day −14	66.95 (60.77–73.14)	61.26 (53.64–68.88)	0.213	Wilcoxon’s test
	Day 0	66.36 (61.32–71.41)	70.36 (63.6–77.12)	0.375	Wilcoxon’s test
Shannon	Day −28	2.579 (2.429–2.729)	2.532 (2.332–2.732)	0.979	Wilcoxon’s test
	Day −14	2.523 (2.328–2.718)	2.546 (2.324–2.769)	0.689	Wilcoxon’s test
	Day 0	2.604 (2.446–2.762)	2.844 (2.648–3.039)	0.048	Wilcoxon’s test
β-Diversity					
UniFrac distance between samples	Day −28	*R*^2^ = 0.00884		0.549	Adonis
	Day −14	*R*^2^ = 0.00954		0.498	Adonis
	Day 0	*R*^2^ = 0.01506		0.024	Adonis

For α-diversity, data are mean (95% CI).

β-Diversity analysis shows the extent of similarity between microbial communities by measuring the degree to which membership or structure is shared between communities^22^. Adonis analysis and PcoA showed significantly different microbial communities between IgA.P and IgA.N groups on day 0 (Fig. 2b, Table 2, *R*^2^ = 0.01506, *p* = 0.024, Adonis). Although no significant differences were observed between IgA.P and IgA.N groups before receiving the last OPV dose (days −28 and −14, Table 2), both α- and β-diversity of gut microbiota showed differences on the day of last OPV dose (day 0). This implied that community richness and bacterial diversity of the bacterial microbiota might reduce the conversion rate of polio-specific IgA.

We found that the phyla *Firmicutes*, *Actinobacteria*, *Proteobacteria*, and *Bacteroidetes* accounted for more than 95% of the gut microbiota, and the overall composition of the bacterial microbiota was broadly similar in each group (Supplementary Table 5). At the time of receiving OPV, the phylum *Firmicutes* was significantly enriched in IgA.N infants, and the phylum *Actinobacteria* was significantly enriched in IgA.P infants (Fig. 2c and Supplementary Table 5; *Firmicutes*: 44.90% [IgA.N] versus 25.31% [IgA.P], *p* < 0.01; *Actinobacteria*: 20.61% [IgA.N] versus 33.08% [IgA.P], *p* < 0.01, Wilcoxon’s test). In addition, for each immunization schedule group (IPV + 2bOPV, 2IPV + bOPV, or 2IPV + tOPV), the relative abundances of the class *Clostridia* were higher in IgA.N, and the class *unidentified_Actinobacteria* showed higher in IgA.P at day 0 (Supplementary Fig. 2 and Supplementary Table 5).

### Impact of specific phylotypes on polio-specific intestinal mucosal IgA response

We analyzed relative abundance of the top 10 phylum-level, and all detected class-level, order-level, family-level, and genus-level members of the gut microbiota between IgA.N and IgA.P infants (36 class-level, 60 order-level, 102 family-level, and 252 genus-level members), we detected the relative abundances of two phylum-level, two class-level, two order-level, three family-level, and one genus-level member of the gut microbiota were significantly different between IgA.N and IgA.P infants (Supplementary Tables 5 and 6).

**Fig. 3:**
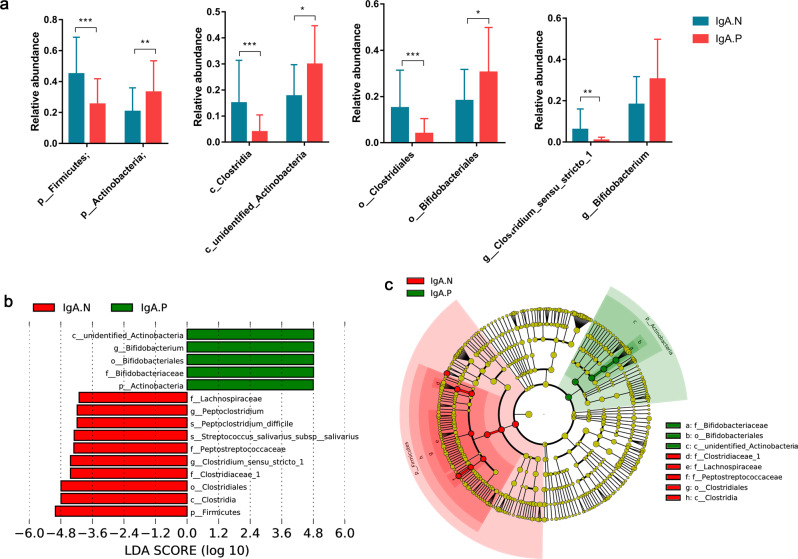
Pivotal phylotypes of the gut microbiota are associated with the poliovirus-specific IgA response. **a** Relative abundance of pivotal phylotypes in class-, order-, family-, and genus-level (presented as the means with SD). Wilcoxon’s test: *p < 0.05; **p < 0.01; ***p < 0.001. **b**, **c** Key phylotypes of the gut microbiota are associated with the poliovirus-specific IgA response identified using LEfSe analysis. **b** The histogram shows the LDA scores computed for features at the OTU level that were differentially abundant between IgA.N and IgA.P infants. **c** The evolutionary branching diagram shows the taxonomic rank of pivotal phylotypes. Red and green nodes show pivotal phylotypes in IgA.N and IgA.P infants, and node size represents the relative abundance of the phylotype. IgA.P: *n* = 66; IgA.N: *n* = 39.

The relative abundance of the phylum *Firmicutes*, class *Clostridia*, order *Clostridiales*, and genus *Clostridium sensu stricto* was significantly higher in IgA.N infants on day 0. In contrast, higher levels of the phylum *Actinobacteria*, class *unidentified Actinobacteria*, order *Bifidobacteriales*, and genus *Bifidobacterium* was observed in IgA.P infants compared with IgA.N infants (Fig. 3a, Supplementary Table 6).

A robust algorithm was used to identify features that were statistically different among biological classes; linear discriminant analysis (LDA) effect size (LEfSe)^23^ was employed to identify specific phylotypes that affected gut mucosal IgA response to the poliovirus vaccine (Fig. 3b). On the day of OPV (day 0), 15 phylotypes were found to be highly effective biomarkers for separating the gut microbiotas of IgA.P and IgA.N infants (Fig. 3b). Five of those phylotypes were higher, and ten of those phylotypes were lower, in the IgA.P group compared with the IgA.N group. Cladograms (Fig. 3c) showed that the predominant phylotypes belonged to the branches *Clostridia* and *unidentified Actinobacteria*, respectively, which were consistent with the above results.

The random forests model predicted the results of IgA.P infants versus IgA.N infants with an accuracy of 82.32%, when the number of variables equaled 100 (Supplementary Fig. 3a). Specifically, there were 39 responders and 66 nonresponders. As such, an uninformative model assigning all individuals as nonresponders would give a baseline accuracy of 66/105 (63%). *Peptoclostridium difficile*, *Peptoniphilus harei*, *Anaerococcus vaginalis*, *Clostridium perfringens*, *Slackia exigua, Solobacterium moorei*, *Clostridium paraputrificum*, *Lactobacillus fermentum*, and *Clostridium tertium*, which belonged to the class *Clostridia*, and *Bifidobacterium breve*, which belonged to the class *unidentified Actinobacteria*, had mean importance scores that far outweighed those of any other species (Supplementary Fig. 3b, c).

## Discussion

Our findings showed that the new routine vaccination schedule reduced the induction probability of anti-polio type 2 mucosal IgA as a result of the lack of type 2 components in bOPV, although the positive conversion rate of anti-polio mucosal IgA in different sequential schedule groups did not show a statistically significant difference. Previous studies suggested that OPV is a much better mucosal immunogen than IPV, since IPV has limited ability to induce mucosal antibody responses^24–26^. However, some studies suggest that bOPV and at least one dose of IPV can provide low intestinal immunity to PV2^3,27,28^. We also detected enteric IgA to type 2 poliovirus in some infants administered IPV + 2bOPV or 2IPV + bOPV, but the schedules did not include type 2 OPV, which is possibly a result of cross-immunity to type 2 poliovirus. Our study provided complementary evidence that the combined IPV/bOPV schedule would induce a degree of primary intestinal mucosal immunity to type 2 poliovirus^28^. In addition, the possibility that a few participants were exposed to a type 2-attenuated strain cannot be excluded, and this is a limitation for our study.

In the context of the current polio immunization strategy, we aimed to elucidate factors affecting vaccine-induced mucosal antibody responses^29,30^.

Some studies reported that low oral vaccine immunogenicity in developing countries is associated with the presence of bacterial and viral enteric pathogens^31–33^. Among infants in Guangxi, China, before they received the last dose of OPV, we detected enrichment of *Clostridia* in those who tested negative for anti-polio mucosal IgA after OPV inoculation; *Clostridia* could be linked with a decreased IgA conversion rate from the poliovirus vaccine, which is a biomarker of mucosal antibody responses. Recently, Atarashi et al.^34,35^ reported that clusters IV, XIVa, and XVIII of *Clostridia* had high potency in enhancing T-regulatory cell abundance and inducing important anti-inflammatory molecules upon inoculation into germ-free mice. These results also indicated that T-regulatory cell induction by *Clostridia* might explain the inhibition of poliovirus vaccine-induced mucosal antibody responses in infants rich in *Clostridia*. However, further studies are needed to confirm this.

Many studies have reported significantly increased vaccine-specific antibodies after administration of probiotics. For example, in a study performed in France, the subjects were supplemented with *Bifidobacterium lactis* or *Lactobacillus acidophilus* before receiving an oral cholera vaccine. They found that the probiotic group exhibited a higher frequency of vaccine-induced IgG compared with the placebo group^36^. Similar to this study, our findings suggest that the genus *Bifidobacterium* may promote better outcomes in polio-specific mucosal IgA response.

In our study, we failed to observe other enteropathogens, such as the genera *Fusobacterium* and *Propionibacterium*, that may have been associated with lower mucosal antibody responses. A study in South India did not observe the inhibitory effects of enteropathogens on rotavirus or OPV immunogenicity. Indeed, infants harboring at least one bacterial pathogen were more likely to respond to this vaccine^37^. This may indicate that not all enteropathogens interfere with the immune response of a vaccine.

We found that the gut microbiota were more diverse in IgA.N infants compared with IgA.P infants at the time of vaccination. In a recent study, Praharaj et al.^19^ found that the microbiota were more diverse in neutralizing antibody nonresponders compared with responders^19^. These studies indicated that intestinal microbiota diversity was a disadvantage in terms of the immune response to OPV, whether it was an IgG or IgA antibody response^19^. Kuss et a.^38^ assessed whether the intestinal microbiota were associated with enteric virus replication, and the results indicated that antibiotic-treated mice were less susceptible to poliovirus infection, supporting minimal viral replication in the intestine. They suggested^38^ that poliovirus was bound to the surface polysaccharides of bacteria, possibly enhancing poliovirus infectivity. The above reports suggested that the diversity of intestinal microbiota was positively correlated with virus replication, and negatively correlated with immune response to OPV. Our results also suggest that the diversity of intestinal microbiota at the time of OPV inoculation is negatively correlated with the mucosal immune response to OPV. However, our understanding of the correlations between live-attenuated vaccine mucosal immunity, proliferation of live-attenuated strains, and the intestinal microbiota is still preliminary, and more research is needed to elucidate these relationships.

However, greater intestinal microbiota diversity may inhibit responses to a particular vaccine, but not to others. For example, in a previous study, volunteers were vaccinated orally with a live-attenuated Salmonella Typhi (*Ty21a*) vaccine, and the volunteers showing higher CD8^+^ interferon-γ levels harbored greater diversity in their intestinal microbiota^39^. These findings suggest that microbiome compositions influence antibody responses, but the effects of diversity of bacterial microbiota is different from above.

Mucosal antibody responses play an important role in inhibiting virus replication, especially in developing regions where mucosal antibody responses are important in blocking fecal–oral transmission^3^. There are many factors that affect antibody responses^4,5,40^. In our study, we focused on the influence of microbiota on mucosal antibody responses to the poliovirus vaccine. In addition, we analyzed the influence of other factors, including sex, race, and feeding. Sex and race failed to show any effect on mucosal antibody responses to poliovirus vaccine. However, interestingly, infants that were breast-fed showed a higher IgA.P rate compared with formula-fed infants. As we known, maternal milk contains a level of poliovirus-specific antibodies; therefore, breast-fed infants may passively acquire maternal antibodies^41,42^. The data of IgA detection at baseline indicates that few infants were positive for polio-specific IgA antibody (Supplementary Table 1), and the infants who were IgA.P at baseline were all breast-fed. However, the slight difference in the positive rate of polio-specific IgA at baseline between breast-fed and formula-fed infants was not statistically significant (5.71%, 4/70 in breast-fed group versus 0.00%, 0/35 in formula-fed group; *p* = 0.299, Pearson’s *χ*^2^ test). Therefore, passive immunity was only partly responsible for the higher positive rate of polio-specific IgA in breast-fed infants after they received OPV, and there must also be other factors. Human milk contains bioactive components that protect the infant from pathogenic infection, facilitate intestinal and immune development, and support healthy gut microbes. An important bioactive component of human milk is human milk oligosaccharides (HMOs), which are a family of soluble glycans that are sialylated or fucosylated. HMOs can modulate an infant’s local and systemic immunity through promoting a healthy microbial environment, stimulating the maturation of gut epithelium and cytokine secretion, and binding monocytes and neutrophils^43^. For the past decade, many studies have shown that human milk contains a higher concentration and a greater structural diversity of fucosylated oligosaccharides compared with the milk oligosaccharides in other species, including cow’s milk from which many infant formulae are derived^44^. This maybe one reason for the higher IgA.P rate observed in breast-fed infants after they received OPV. In addition, human milk contains cytokines that might impact IgA secretion^45^. For example, interleukin-6 is known to enhance IgA production, tumor necrosis factor (TNF-α) enhances secretory component production, and TNF-β can trigger isotype switching toward IgA-producing B cells. These factors may be responsible for the higher polio-specific IgA.P rate in breast-fed infants after these infants received OPV.

Our study had limitations. Some other factors, such as genetic factors, have been attributed to mucosal antibody responses^46–48^. Unfortunately, in this study, individual genetic differences were not examined because of restrictions imposed by the sampling requirements. Because of the limitation of clinical trial protocol, only the first 10% of paticipants’ stool samples were collected, giving only a modest sample size. Furthermore, the stool samples were pooled across three different schedules and for three different serotypes of IgA. Because of the cross-reactivity among different polio serotypes, the effects of a single serotype of polio vaccine cannot be analyzed clearly. Type 2-specific IgA interferes with the detection of type 1- and type 3-specific IgA, possibly resulting in a higher level of type 1- and type 3-specific IgA detected in the 2IPV + tOPV group. This may explain why we failed to observe any improvement in type 1-specific IgA levels when the competing type 2 OPV was removed. Moreover, we only detected a difference in the microbiota composition between IgA.P and IgA.N infants when the last dose was administered.

In our study, we identified the bacterial microbiota before the last dose of OPV was administered (day 0) using 16S ribosomal RNA sequencing, and measured the IgA antibody levels after the last dose was administered. The anti-polio mucosal IgA conversion rate after OPV inoculation was associated with the status of the intestinal bacterial microbiota at the time of OPV inoculation, indicating the intestinal microbiota may play a specific role during the intestinal immune response process, which was worth further study.

## Methods

### Experimental design

Clinical trials, entitled “Randomized, Double-Blind, Single-Center, Parallel Trial to Evaluate the Safety and Immunogenicity by Different Sequential Immunization Schedules of Bivalent Oral Poliomyelitis Vaccine Co-administered with IPV in Infants Aged 2 Months,” were conducted in Guangxi Province, China, during 2015-2016. The clinical trial protocol was verified and approved by the ethics committee of the Guangxi Zhuang Autonomous Region, Registered in 2015 (ClinicalTrials.gov ID: NCT03614702).

### Participants

Infants who had not yet received basic vaccinations against polio were recruited. Healthy 2-month-old infants from full-term pregnancies with birth weights over 2.5 kg and no medical defects were selected according to the inclusion/exclusion criteria, and the qualified infants were included as participants in the study. Other inclusion/exclusion criteria were consistent with the typical criteria used in clinical vaccine studies, and infants with any factors that interfered with the postvaccination immune response or allergies to vaccine components were excluded. The inclusion criteria were as follows: guardians are informed, agree, and sign informed consent; guardians and the family follow the requirements of the clinical trial protocol; no immune globulin immunization history after birth (except hepatitis B immune globulin), no other live vaccination history 28 days before vaccination, and no inactivated vaccination history 14 days before vaccination; axillary temperature <37.1°C. The exclusion criteria were as follows: allergy, convulsions, epilepsy, encephalopathy, or psychosis history or family history; allergy to neomycin, streptomycin, or polymyxin B; immunodeficiency or receiving immunosuppressors; poliomyelitis history; acute febrile disease or infectious disease; abnormal stage of labor, asphyxiation history, congenital malformation, developmental disorder, or severe chronic disease; severe anaphylactic reactions following previous vaccination; administration of oral steroids for 14 days consecutively within 1 month before the trial; fever in the past 3 days (axillary temperature ≥38.0 °C); diarrhea within the past week (defecation frequency ≥3 times/day); participation in other drug clinical trials; OPV vaccination contraindications or other conditions that may influence evaluation.

The guardians and their families needed to be willing to voluntarily comply with the requirements of the clinical trial protocol, and an informed consent form had to be signed by both the guardians of the participants and the research doctor prior to the study. Participants could voluntarily withdraw at any time during the trial. Withdrawal might also be recommended in the following instances: failure to adhere to the follow-up visits; violation of or deviation from the trial protocol; and other abnormal symptoms that affected the trial.

### Blinding and randomization

The vaccines were coded in blind tests. The code was adhered to the vaccine bottle and the large package of sugar pills, completely covering the original label. The investigators and participants were blinded to the vaccine they were receiving (tOPV versus bOPV). However, it was not possible for IPV and OPV to be blinded due to their different appearances and vaccination routes.

A random allocation form was used to randomly group the subjects. The form is a scratch card. The research number is printed on it, and each research number corresponds to three vaccine codes. Each vaccine code is covered by a code film. At each vaccination, the film is scraped to obtain the vaccine code.

In practice, the subjects obtained their research number and vaccine code randomly. Based on the sequential order of arrival, the investigators filled the screening number and initials into the corresponding columns to obtain the research number. Then, the code film in the scratch card was scraped; note, number jumping was forbidden. The grouping results (research number and vaccine code) were recorded in the original notebook and vaccination card. The subjects, carrying their original notebook and vaccination card, were vaccinated.

The test vaccine was labeled according to the code, and the investigators in the vaccination group vaccinated the subjects based on the code in the original notebook and vaccination card. Furthermore, the research number and name of the subjects were recorded on the vaccine label before vaccination. The vaccine label was adhered to the original notebook, and the vaccination card was handed in after vaccination. The vaccine was then checked based on the vaccination cards after each day, and the cards were sealed for safekeeping by the vaccination group. At each vaccination, a new vaccination card was initiated. All of the used vaccination cards were kept by the vaccination group, so that other investigators could not review them.

### Stool samples

Before the clinical trial was finished, all participants and laboratory researchers remained blind. Stool samples were labeled with a research number rather than any other grouping information. When the clinical trial was finished, the data were locked, and the statistician performed analyses based on the vaccine codes.

A total of 1200 qualified 2-month-old infants who had not received basic vaccinations against polio were recruited. The informed consent form was signed by both guardians of the infant, as well as the research doctor before the study. The first, second, and third vaccine doses were inoculated at 2, 3, and 4 months of age, according to different sequential immunization schedules (IPV-bOPV-bOPV, IPV-IPV-bOPV, or IPV-IPV-tOPV), respectively. Fecal samples from the first 10% of participants enrolled clinical trial were collected. Because the research numbers and vaccine codes were randomly assigned to each infant, and the investigators, laboratory researchers, and infants remained blind to the research numbers and vaccine codes, the first 10% of participants enrolled in the clinical trial were randomly assigned to an immune schedule. The fecal samples of 14 days after the last dose and before the second dose were collected to detect IgA antibodies. At the same time, bacterial microbiota from this 10% of participants were identified using 16S ribosomal RNA sequencing 28 days before, 14 days before, and at the last dose of OPV. All of the stool samples were transported by cold chain transporters and stored at −80 °C until analysis.

### Analysis of IgA in stool samples

Stool samples were homogenized, and then 1 g of the sample was added to 5 ml of PBS (pH 7.4) and evenly mixed. After centrifuging for 20 min at 800–1000 × *g*, the supernatants were harvested and stored for future use. An aliquot of the supernatant was used in an enzyme-linked immunosorbent assay (ELISA), in which each type of polio-specific IgA in the stool samples were measured using a human poliovirus type 1, 2, and 3 antibody IgA ELISA Kit, according to the manufacturer’s instructions (SunLong Biotech, Hangzhou, China).

Stool samples were weighed, and bacterial DNA was isolated from the stool samples using a QIAamp Stool Mini Kit (Qiagen). Phusion^®^ High-Fidelity PCR Master Mix with GC Buffer (New England Biolabs) was used for 16S rRNA polymerase chain reaction (PCR). Using DNA as the template, the hypervariable V4 region of the 16S rRNA gene was amplified using the primer pair 338 F: ACTCCTACGGGAGGCAGCA and 806 R: GGACTACHVGGGTWTCTAAT, and the reverse primer contained a 6-nucleotide barcode^49^. PCRs were run using the following program: 5 min of denaturation at 95 °C, 30 cycles at 95 °C for 30 s (denaturation), 58 °C for 30 s (annealing), and 72 °C for 30 s (elongation), and a final extension step at 72 °C for 10 min. QiagenGel Extraction Kit (Qiagen) was used for purification of products. The resulting amplicons were purified, quantified, pooled, and sequenced on an Illumina MiSeq PE300 platform (Novogene Bioinformatics Technology Co., Ltd.). Reactions were run, and the 16s rRNA gene was analyzed to determine bacterial compositions and diversity using the Illumina MiSeq platform. Analysis of IgA, the DNA extractions, and PCR assays were randomized and were not batched by the study group or vaccine response. The experimenter remained blind for sample information.

### Data analysis

Unblinding regulations were such that when the clinical trial was finished, the data were locked, the statistician performed analyses based on the vaccine codes.

Paired-end reads was assigned to samples based on their unique barcode and truncated by cutting off the barcode and primer sequence. Paired-end reads were merged using FLASH^50^, a very fast and accurate analysis tool, which was designed to merge paired-end reads when at least some of the reads overlap the read generated from the opposite end of the same DNA fragment, and the splicing sequences were called raw tags. Quality filtering on the raw tags^51^ was performed under specific filtering conditions to obtain the high-quality clean tags according to the QIIME^52^ quality-controlled process. The procedure was as follows: (1) *intercepting tags*: raw tags are truncated from the first low-quality base point of a continuous low-quality value (the default quality threshold was ≤19) to the set length (the default length was 3); (2) t*ags’ length filtration*: after the tags has been intercepted, the data set of the tags is obtained, and the tags with a continuous high-quality base length of <75% of the tag length were further filtered out. Tags obtained after QIIME processing needed to be processed to remove chimeric sequences. The tags were compared with the reference database (Gold database) using the UCHIME algorithm^53^ to remove the chimeric sequences^54^, then the effective tags were finally obtained. Sequences analyses were performed using the Uparse software^55^ (Uparse v7.0.1001). Sequences with ≥97% similarity were assigned to the same operational taxonomic units (OTUs). Representative sequences for each OTU were screened for further annotation. Species annotation was performed on the OTUs of representative sequence, and species annotation analysis was performed using the SSUrRNA database of SILVA and the Mothur method (the threshold was set to 0.8–1). Taxonomic information was obtained and the community composition of each sample was counted at each taxonomic level. For taxonomic comparisons, Wilcoxon’s rank-sum tests were used and *p* values were adjusted via Benjamini–Hochberg false discovery rate correction. All statistical tests were considered significant at *p* < 0.05, two sided.

The α- and β-diversity was determined using QIIME^52^. To compute the α-diversity, we rarified the OTU table and calculated three metrics: observed species (an estimate of the number of unique OTUs in each sample), the PD whole tree, and the Shannon index. We compare α-diversity among different groups using Wilcoxon’s rank-sum test. QIIME calculates both the weighted and unweighted UniFrac, which are phylogenetic measures of β-diversity. We used weighted UniFrac values for PCoA. PCoA helps to obtain and visualize principal coordinates from complex, multidimensional data. It transforms a distance matrix into a new set of orthogonal axes. The maximum variation factor is demonstrated by the first principal coordinate, the second maximum variation factor by the second principal coordinate, and so on. To mine deeper data on microbial diversity and to determine the significance of differences between the samples, statistical analysis methods, including the LEfSe and Adonis, were used.

LEfSe^23^ is an algorithm for high-dimensional biomarker discovery that can identify genomic features that characterize the differences between two or more biological conditions. LEfSe emphasizes both statistical significance and biological relevance, allowing researchers to identify differentially abundant features that are also consistent with biologically meaningful categories. The differential features were identified at the OTU level. LEfSe analysis was performed under the following conditions: (1) the *α* value for the factorial Kruskal–Wallis test among classes was <0.05, and (2) the threshold for the logarithmic LDA score for discriminative features was >3.0.

Random forest modeling^56^ of gut microbiota development was performed with the “randomForest” package in R. All receiver-operating curves presented for random forest models are based on the out-of-bag error rates. For each model, 10-fold cross-validations were performed to further estimate the generalization error of the model. The rfcv function was used for selecting reduced models. The above data analysis about gut microbiota was conducted by Novogene Bioinformatics Technology Co., Ltd.

We compared positive conversion rates of polio-specific gut mucosal IgA among different groups, using the Pearson’s *χ*^2^ test performed by SPSSV22.0 (IBM, USA). All statistical tests were considered significant at *p* < 0.05, two sided.

### Reporting summary

Further information on experimental design is available in the Nature Research Reporting Summary linked to this article.

## Supplementary information

Supplementary file

Reporting Summary

## Data Availability

The sequence data were deposited in the NCBI Sequence Read Archive, accession number: PRJNA557087.
