# Supplementary material for: Influence of gut microbiota on mucosal IgA antibody response to the polio vaccine
Source: NPJ Vaccines. 2020 Jun 9;5:47. doi: 10.1038/s41541-020-0194-5 (PMC7283253; doi:10.1038/s41541-020-0194-5)
Supplement: Supplementary file 1 — Supplementary file [file 41541_2020_194_MOESM1_ESM.pdf]

Supplementary Information

Supplementary Figures

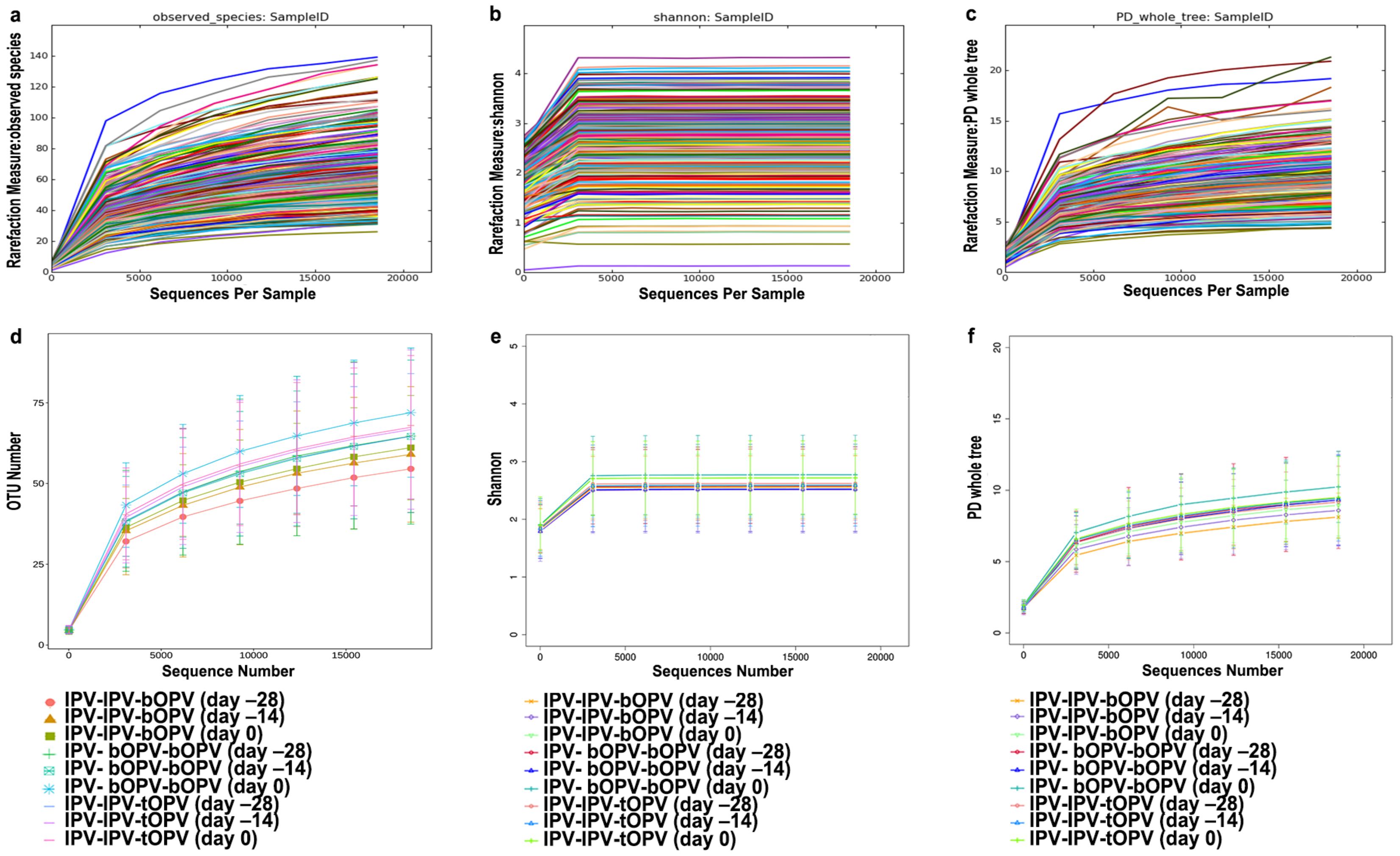

Supplementary Figure 1. Diversity of gut microbiota. (**a**) Rarefaction analysis of sampling by observed OTU method. (**b**) Shannon Diversity Index curves, which indicate the diversity of taxa in an individual sample. (**c**) Rarefaction analysis of sampling by PD whole tree method. (**d**) Rarefaction analysis of nine groups by observed OTU method. (**e**) Shannon Diversity Index curves, which indicate the diversity of taxa in nine groups. (**f**) Rarefaction analysis of 9 groups by PD whole tree method. Data in (**d**), (**e**) and (**f**) are shown as mean ± standard deviation. Nine groups: IPV-IPV-bOPV (day –28), n=34; IPV- bOPV-bOPV (day –28), n=34; IPV-IPV-tOPV (day –28), n=37; IPV-IPV-bOPV (day –14), n=33; IPV- bOPV-bOPV (day –14), n=34; IPV-IPV-tOPV (day –14), n=36; IPV-IPV-bOPV (day 0), n=34; IPV- bOPV-bOPV (day 0), n=34; IPV-IPV-tOPV (day 0), n=37.

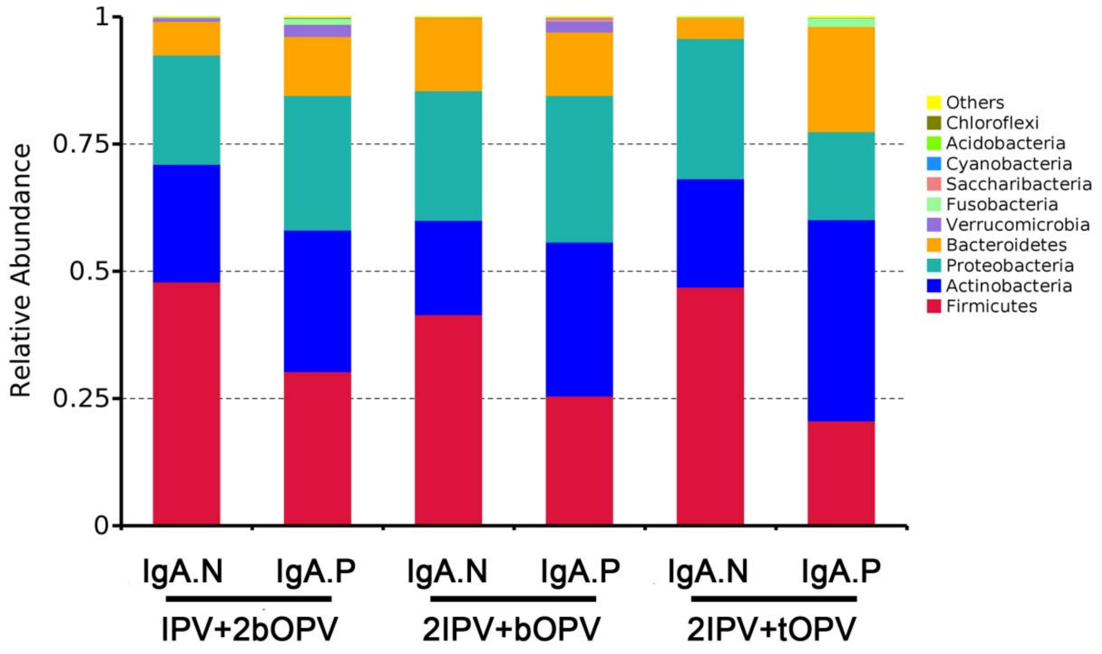

Supplementary Figure 2. Phylum-level compositions of the bacterial microbiota from IgA.N or IgA.P groups of different immunization schedules at the third vaccine dose. IgA.P: n=66; IgA.N: n=39.

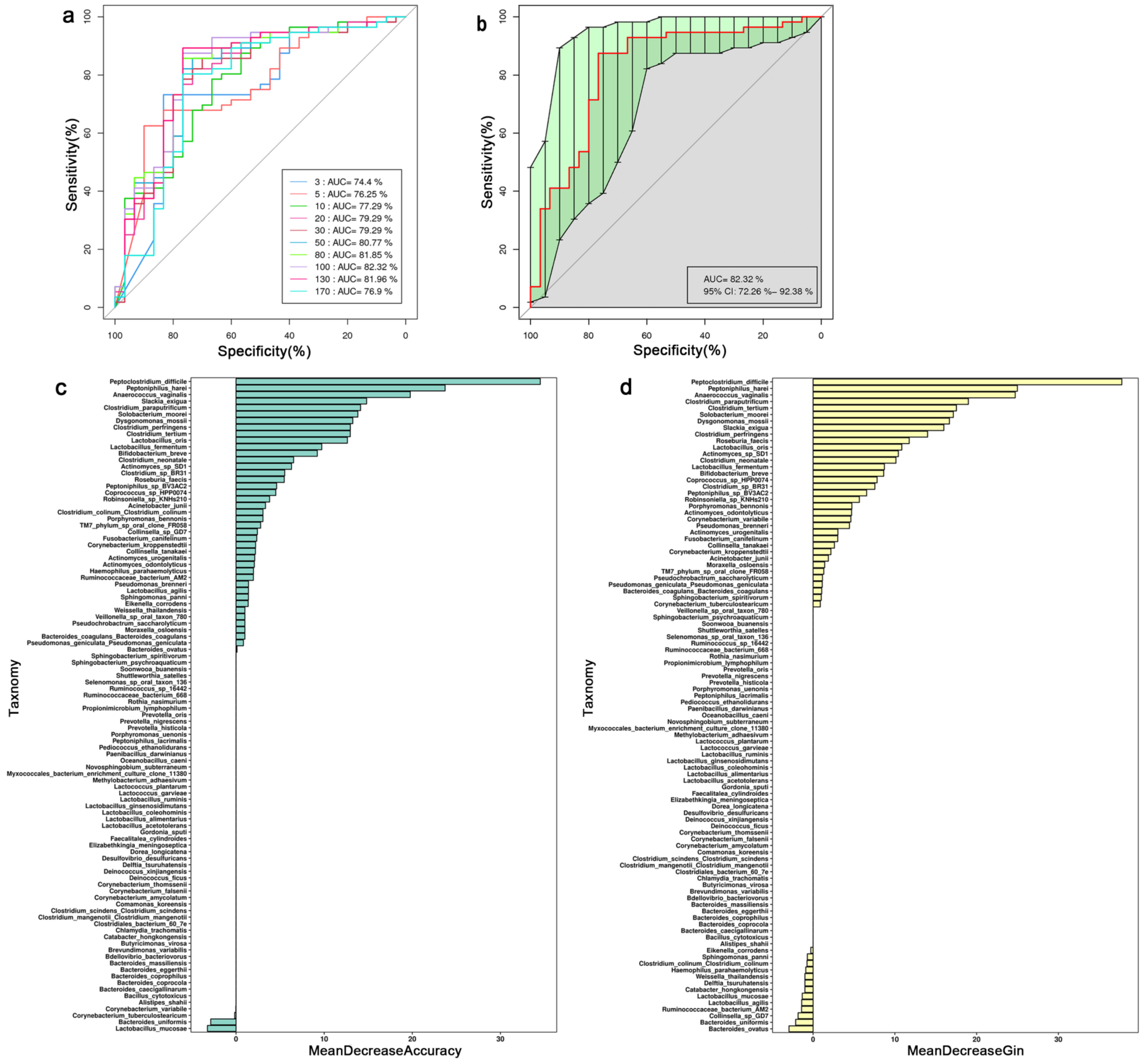

Supplementary Figure 3. Random forest feature selection for detecting poliovirus-specific IgA-positive infants.

(a) Receiver operating characteristic (ROC) curves for the random forest prediction model for distinguishing IgA.P-positive from IgA-negative infants, when 3,5,10,20,30,50,80,100,130,170 important variables were selected respectively to construct random forest models. **(b)** Receiver operating characteristic (ROC) curves for the random forest prediction model(important variables=100). (**c and d)** The highest-ranking corresponding species according to the random forest importance score (Mean Decrease Accuracy and Mean Decrease Gin) for the model predicting poliovirus-specific IgA-positive infants.

Supplementary Tables

Supplementary table 1. The baseline characteristics of infants in each group

	IPV+2bOPV (n=36)	2IPV+bOPV (n=34)	2IPV+tOPV (n=37)	p Value (Pearson CHI-SQUARE)
**Sex**				0.267
**Male**	18/36 (50%)	22/34 (64.7%)	17/37 (45.9%)	
**Female**	18/36 (50%)	12/34 (35.3%)	20/37 (54.1%)	
**Race**				0.348
**Han**	9/36 (25%)	14/34 (41.2%)	11/37 (29.7%)	
**Minorities**	27/36 (75%)	20/34 (58.8%)	26/37 (70.3%)	
**Feeding**				0.294

**Breast fed**	26/36 (72.22%)	19/34 (55.9%)	26/37 (70.27%)	
**Formula fed**	10/36 (27.78%)	15/34 (44.1%)	11/37 (29.73%)	
**IgA positive rate**				
**PV1-IgA**	0/36 (0%)	0/34 (0%)	0/35 (0%)	1
**PV2-IgA**	1/36 (2.78%)	2/34 (5.88%)	0/35 (0%)	0.317
**PV3-IgA**	1/36 (2.78%)	0/34 (0%)	0/35 (0%)	1

Data are n(%).

Supplementary Table 2. Alpha and beta diversity of gut microbiota in breast-fed and formula-fed infants

	Days before Vaccination	Breast fed (n=70)	Formula fed (n=35)	p Value	Test Method
**Alpha diversity**					
**Observed_species**	day-28	58.31(52.75-63.88)	63.26(57.64-68.88)	0.5587	Wilcoxon test
	day-14	62.31(55.48-69.15)	66.16(59.77-72.55)	0.5845	Wilcoxon test
	day0	66.94(60.3-73.59)	68.3(63.2-73.4)	0.865	Wilcoxon test
**Shannon**	day-28	2.579(2.429-2.729)	2.532(2.332-2.732)	0.2577	Wilcoxon test
	day-14	2.523(2.328-2.718)	2.546(2.324-2.769)	0.5943	Wilcoxon test
	day0	2.604(2.446-2.762)	2.844(2.648-3.039)	0.1467	Wilcoxon test
**Beta diversity**					
**UniFrac distance between samples**	day-28	R^2=0.00695		0.613	Adonis
	day-14	R^2=0.00563		0.712	Adonis
	day0	R^2=0.00209		0.976	Adonis

Supplementary Table 3. Relative abundance of gut microbiota in class-, oder-, family- and genus-level between breast fed and formula fed (which have significant difference between IgA.N and IgA.P)

Taxa	Avg(Breast)	Sd(Breast)	Avg(Formula)	Sd(Formula)	FDR-corrected p.value,(Wilcoxon test)
**Phylum**					
**k__Bacteria;p__Firmicutes;**	0.334363415	0.229133954	0.308885198	0.190199804	1
**k__Bacteria;p__Actinobacteria;**	0.283048627	0.199397849	0.287353322	0.189522818	1
**k__Bacteria;p__Proteobacteria;**	0.239794082	0.212232333	0.239793588	0.212465481	1
**k__Bacteria;p__Bacteroidetes;**	0.130564865	0.224340776	0.132851903	0.197536607	1
**Class**					
**k__Bacteria;p__Actinobacteria;c__unidentified_Actinobacteria;**	0.267144637	0.192268131	0.274495092	0.186725611	1
**k__Bacteria;p__Firmicutes;c__Clostridia;**	0.069901243	0.114679365	0.099950572	0.14106059	1
**Order**					

**k__Bacteria;p__Firmicutes;c__Clostridia;o__ Clostridiales;**	0.069900748	0.114679665	0.099950572	0.14106059	0.619123
**k__Bacteria;p__Actinobacteria;c__unidentified_Actinobacteria;o__Bifidobacteriales;**	0.254372905	0.184120289	0.266671609	0.186353767	0.732478
**Family**					
**k__Bacteria;p__Firmicutes;c__Clostridia;o__ Clostridiales;f__Clostridiaceae_1;**	0.021146831	0.05268386	0.038709135	0.088350966	0.755154
**k__Bacteria;p__Actinobacteria;c__unidentified_Actinobacteria;o__Bifidobacteriales;f__Bifidobacteriaceae;**	0.254372905	0.184120289	0.266671609	0.186353767	0.868298
**k__Bacteria;p__Firmicutes;c__Clostridia;o__ Clostridiales;f__Peptostreptococcaceae;**	0.01431636	0.047760222	0.030448708	0.068664603	0.755154
**Genus**					

**k__Bacteria;p__Firmicutes;c__Clostridia;o__Clostridiales;f__Clostridiaceae_1;g__Clostridium_sensu_stricto_1;**	0.021137934	0.052685914	0.038708147	0.088351411	0.895759
**k__Bacteria;p__Actinobacteria;c__unidentified_Actinobacteria;o__Bifidobacteriales;f__Bifidobacteriaceae;g__Bifidobacterium;**	0.254367963	0.18411413	0.266668644	0.186354133	1

Supplementary Table 4. Alpha and beta diversity of gut microbiota in different immunization schedules

	Days before Vaccination	2IPV+bOPV	IPV+2bOPV	2IPV+tOPV	P Value	Test Method
**Alpha diversity**						
**Observed_ species**	day-28	51.91(47.14-56.68)	62.09(53.87-70.31)	62.19(55.91-68.47)	0.023(2IPV+bOPV Vs 2IPV+tOPV);<br>0.0946(2IPV+bOPV Vs IPV+2bOPV);<br>0.5674(2IPV+tOPV Vs IPV+2bOPV)	Wilcoxon test
	day-14	57.06(49.53-64.59)	62.09(53.87-70.31)	65.03(56.61-73.44)	0.1921(2IPV+bOPV Vs 2IPV+tOPV);<br>0.3424(2IPV+bOPV Vs IPV+2bOPV);<br>0.7297(2IPV+tOPV Vs IPV+2bOPV)	Wilcoxon test
	day0	59.71(53.96-65.45)	69(62.15-75.85)	65.62(58.36-72.89)	0.3083(2IPV+bOPV Vs 2IPV+tOPV);<br>0.0709(2IPV+bOPV Vs IPV+2bOPV);<br>0.4074(2IPV+tOPV Vs IPV+2bOPV)	Wilcoxon test

**Shannon**	day-28	2.532(2.337-2.726)	2.55(2.325-2.774)	2.599(2.386-2.811)	0.576(2IPV+bOPV Vs 2IPV+tOPV); 0.9915(2IPV+bOPV Vs IPV+2bOPV); 0.5686(2IPV+tOPV Vs IPV+2bOPV)	Wilcoxon test
	day-14	2.5(2.225-2.775)	2.498(2.234-2.762)	2.592(2.349-2.836)	0.9115(2IPV+bOPV Vs 2IPV+tOPV); 0.7884(2IPV+bOPV Vs IPV+2bOPV); 0.8711(2IPV+tOPV Vs IPV+2bOPV)	Wilcoxon test
	day0	2.586(2.393-2.779)	2.772(2.529-3.014)	2.718(2.5-2.936)	0.3292(2IPV+bOPV Vs 2IPV+tOPV); 0.2676(2IPV+bOPV Vs IPV+2bOPV); 0.8758(2IPV+tOPV Vs IPV+2bOPV)	Wilcoxon test
**Beta diversity**						
**UniFrac distance**	day-28	R^2=0.00703(2IPV+bOPV Vs 2IPV+tOPV); 0.00353(2IPV+bOPV Vs IPV+2bOPV); 0.01673(2IPV+tOPV Vs IPV+2bOPV)			0.023(2IPV+bOPV Vs 2IPV+tOPV); 0.944(2IPV+bOPV Vs IPV+2bOPV);	Adonis

**between samples**			0.304(2IPV+tOPV Vs IPV+2bOPV)	
	day-14	R^2=0.02522(2IPV+bOPV Vs 2IPV+tOPV); 0.01963(2IPV+bOPV Vs IPV+2bOPV); 0.01331(2IPV+tOPV Vs IPV+2bOPV)	0.243(2IPV+bOPV Vs 2IPV+tOPV); 0.252(2IPV+bOPV Vs IPV+2bOPV); 0.439(2IPV+tOPV Vs IPV+2bOPV)	Adonis
	day0	R^2=0.01558(2IPV+bOPV Vs 2IPV+tOPV); 0.01276(2IPV+bOPV Vs IPV+2bOPV); 0.01331(2IPV+tOPV Vs IPV+2bOPV)	0.336(2IPV+bOPV Vs 2IPV+tOPV); 0.502(2IPV+bOPV Vs IPV+2bOPV); 0.196(2IPV+tOPV Vs IPV+2bOPV)	Adonis

Supplementary Table 5. Relative abundance of gut microbiota in phylum-level and class-level from IgA.N or IgA.P groups (The top 10 phylum-level and all detected 36 class-level members were analyzed)

Taxa	Avg(IgA.N)	Sd(IgA.N)	Avg(IgA.P)	Sd(IgA.P)	FDR-corrected p.value,(Wilcoxon test)
**Phylum**					
**Total**					
**k__Bacteria;p__Firmicutes;**	0.44896547	0.237625754	0.25308042	0.165502293	0.000188342
**k__Bacteria;p__Actinobacteria;**	0.206097883	0.153462956	0.330811226	0.203439407	0.008713709
**k__Bacteria;p__Proteobacteria;**	0.249274737	0.235927446	0.234259766	0.196999735	0.690267132
**k__Bacteria;p__Bacteroidetes;**	0.092695209	0.172047847	0.15414263	0.234788281	0.390750513
**2IPV+bOPV**					
**k__Bacteria;p__Firmicutes;**	0.415814904	0.25040933	0.255461662	0.193828382	0.287851552
**k__Bacteria;p__Actinobacteria;**	0.185256872	0.13027418	0.303129846	0.146490266	0.133909567
**k__Bacteria;p__Proteobacteria;**	0.254232355	0.245904776	0.287484685	0.238750653	0.630722125

**k__Bacteria;p__Bacteroidetes;**	0.14359275	0.231310752	0.124851934	0.23830195	0.630722125
**2IPV+tOPV**					
**k__Bacteria;p__Firmicutes;**	0.46951768	0.190117369	0.206862181	0.149933529	0.000550843
**k__Bacteria;p__Actinobacteria;**	0.212894987	0.149294052	0.395575499	0.24137453	0.018024404
**k__Bacteria;p__Proteobacteria;**	0.275982788	0.240510531	0.172817425	0.150439691	0.065661136
**k__Bacteria;p__Bacteroidetes;**	0.040607445	0.096801368	0.206653253	0.272617165	0.018024404
**IPV+2bOPV**					
**k__Bacteria;p__Firmicutes;**	0.479608206	0.273508519	0.303550529	0.151282668	0.554358392
**k__Bacteria;p__Actinobacteria;**	0.231437271	0.196320896	0.278094189	0.178782175	0.781649019
**k__Bacteria;p__Proteobacteria;**	0.214939513	0.23406162	0.264379176	0.199662338	0.781649019
**k__Bacteria;p__Bacteroidetes;**	0.066220645	0.096653921	0.116436955	0.178071114	1
**Class**					
**Total**					
**k__Bacteria;p__Actinobacteria;c__**	0.1762528	0.1211282	0.29826554	0.14875755	0.022593

**unidentified_Actinobacteria;**					
**k__Bacteria;p__Firmicutes;c__Clostridia;**	0.1498184	0.1640202	0.03862163	0.06562775	2.22E-05
**IPV+2bOPV**					
**k__Bacteria;p__Actinobacteria;c__unidentified_Actinobacteria;**	0.2118696	0.1800827	0.264838	0.1770491	0.362941
**k__Bacteria;p__Firmicutes;c__Clostridia;**	0.1892917	0.16727	0.04354896	0.06436669	0.009919
**2IPV+bOPV**					
**k__Bacteria;p__Actinobacteria;c__unidentified_Actinobacteria;**	0.1762528	0.1211282	0.29826554	0.14875755	0.022593
**k__Bacteria;p__Firmicutes;c__Clostridia;**	0.136105	0.179272	0.01857392	0.02141924	0.016191
**2IPV+tOPV**					
**k__Bacteria;p__Actinobacteria;c__unidentified_Actinobacteria;**	0.189342	0.1239058	0.37443376	0.23875597	-0.17596
**k__Bacteria;p__Firmicutes;c__Clostridia;**	0.1314819	0.1430246	0.0474162	0.08293812	-0.17596

The samples were collected at the day of last dose of OPV(Day 0); IgA.P: n=66; IgA.N: n=39.

Supplementary Table 6. Relative abundance of gut microbiota in class-, oder-, family- and genus-level which have significant difference between IgA.N and IgA.P (P<0.05). (All detected members were analyzed, including 36 class-level, 60 order-level, 102 family-level and 252 genus-level members, and only the species with differences in relative abundance were shown in the table)

Taxa	Avg(IgA.N)	Sd(IgA.N)	Avg(IgA.P)	Sd(IgA.P)	FDR-corrected p.value,(Wilcoxon test)
**Class**					
**k__Bacteria;p__Actinobacteria;c__unidentified_Actinobacteria;**	0.176252814	0.1211282	0.29826554	0.14875755	0.006797
**k__Bacteria;p__Firmicutes;c__Clostridia;**	0.149818396	0.1640202	0.03862163	0.06562775	2.22E-05
**Order**					
**k__Bacteria;p__Firmicutes;c__Clostridia;o__Clostridiales;**	0.149817509	0.164021	0.03862163	0.06562775	7.73E-05
**k__Bacteria;p__Actinobacteria;c__unidentified_Actinobacteria;o__Bifidobacteriales;**	0.181495735	0.1357878	0.30396449	0.19439068	0.023376
**Family**					

**k__Bacteria;p__Firmicutes;c__Clostridia;o__Clostridiales;f__Clostridiaceae_1;**	0.059922213	0.1001101	0.00755322	0.01568595	0.001293
**k__Bacteria;p__Actinobacteria;c__unidentified_Actinobacteria;o__Bifidobacteriales;f__Bifidobacteriaceae;**	0.181495735	0.1357878	0.30396449	0.19439068	0.023421
**k__Bacteria;p__Firmicutes;c__Clostridia;o__Clostridiales;f__Peptostreptococcaceae;**	0.043782792	0.0826305	0.00545731	0.02093508	0.023421
**Genus**					
**k__Bacteria;p__Firmicutes;c__Clostridia;o__Clostridiales;f__Clostridiaceae_1;g__Clostridium_sensu_stricto_1;**	0.059913341	0.1001142	0.00754902	0.01568654	0.002726
**k__Bacteria;p__Actinobacteria;c__unidentified_Actinobacteria;o__Bifidobacteriales;f__Bifidobacteriaceae;g__Bifidobacterium;**	0.181494848	0.1357874	0.30395873	0.19438657	0.06711

The samples were collected at the day of last dose of OPV(Day 0); IgA.P: n=66; IgA.N: n=39.
